# Supplementary material for: General molten-salt route to three-dimensional porous transition metal nitrides as sensitive and stable Raman substrates
Source: Nat Commun. 2021 Mar 2;12:1376. doi: 10.1038/s41467-021-21693-4 (PMC7925654; doi:10.1038/s41467-021-21693-4)
Supplement: Supplementary file 1 — supplementary information [file 41467_2021_21693_MOESM1_ESM.pdf]

General molten-salt route to three-dimensional porous transition metal nitrides as sensitive and stable Raman substrates

(1) Supplementary methods

Adsorption experiment

During the adsorption test, 50 mg of the 3D porous TMNs were added into the solvent to be adsorbed (100 mL) at room temperature and one atmosphere. After 10 min of full contact, the 3D porous TMNs were quickly separated by vacuum filtration. Weight measurements should be made as soon as possible to avoid evaporation of organic liquids with low boiling points. The weight of 3D porous TMNs before and after adsorption was recorded, and the weight increment was calculated.

Photothermal Test

50 mg of 3D porous VN was dispersed well in 10 mL distilled water, under the assistance of ultrasonic bath. The dispersed mixture was deposited on the foam rubber membrane under vacuum. The formed VN/foam rubber film was placed on a heat plate and the temperature was kept at 70 °C for 12 min. Foam rubber was chosen as a bottom supporting layer because of its unique inner microporous structure and hydrophilicity. The microporous structure of the cellulose membrane enables efficient absorption of water through capillary effect. This effect enables more rapid replenishment of surface water after evaporation, while the hydrophilicity would benefit the water adhesion and speed up the water transfer upward. Under illumination of a solar simulator at power density of 2 KW m^{-2}, the light-thermal

system can be quickly heated up in 10 s and generates visible steam flow on top of the water surface.

Hydrophobicity Measurement

The contact angle was measured using a contact - angle measurement system (Contact Angle System OCA 20, Dataphysics). For the test of hydrophobic property, a water droplet was placed on the top by a syringe needle. The droplet keeps a round shape and merges into the structure. The picture of the formed droplet was taken by an optical microscope from the side view and was then inserted into an "Image Software". The contact angle was calculated by the Software for different substrates.

Enhanced Factor Calculation

To calculate the EF of the 3D porous VN samples, the ratio of SERS to normal Raman spectra (NRS) of R6G was determined by using the following calculating Formula 1

$$EF = (I_{SERS}/N_{SERS})/(I_{NRS}/N_{NRS}) \quad (1)$$

$$N_{SERS} = N_A n S_{Irr}/S_{dif} \quad (2)$$

$$N_{NRS} = dS_{Irr}hN_A/M \quad (3)$$

where I_{SERS} and I_{NRS} refer to the peak intensities of the SERS and NRS, respectively. N_{SERS} and N_{NRS} correspond to the number of probe molecules excited in the SERS and NRS tests. In the SERS measurements, two Raman scattering peaks, R_1 at 612 cm^{-3} and R_2 at 773 cm^{-3} were selected for the calculations of the EF. For comparison, the peak intensities of the R6G (1×10^{-2} M, aqueous solution) directly placed on bare glass were detected as NRS data. To decrease the measuring error, the intensities were

obtained by continually ran the test procedure at randomly selected 10 points and took the average. N_{SERS} is calculated by formula 2, where N_A refer to the Avogadro's constant, n correspond to the molar quantity of the probe molecule, S_{Irr} refer to the irradiation area under the laser beam (5 μm in diameter), and S_{dif} refer to the diffusion area of the substance to be tested on the substrate. In a typical test, one drop (20 microliter) of the probe solution was dropped onto the SERS substrate, and the probe solution was spread into a circle with a diameter of 4 mm when the solution is completely dry. N_{NRS} is determined by the formula 3, where d is the packing density of R6G molecules in the surface of substrate (1.4×10^{21} molecule/cm^3), h refer to the laser confocal depth (26 μm), M correspond to the molecule weight of R6G (479).

DFT computational details

All the periodic Density functional theory (DFT) calculations were carried out using the Vienna ab initio simulation package (VASP)[1-2]. The generalized gradient approximation of Perdew-Burke-Ernzerhof (GGA-PBE)[3-4] was used to describe exchange-correlation functional and the cutoff energy was set to 600 eV for the plane-wave basis to expand the one-electron wave function, and the Brillouin zone (BZ) integrations were performed using a Monkhorst-Pack theme[5] with k-point mesh of 7 × 7 × 1. Geometry optimization were stopped until the forces on each atom were less than 0.01 eV/Å and the convergence criteria of electronic self-consistent energy was set to 1×10^{-5} eV. The optimized lattice constant of VN bulk is of bulk Pd is 4.12 Å, in good agreement with the experimental result of 4.14 Å[6].

Calculation of photothermal conversion efficiency

According to the Beer-Lambert Law, the mass extinction coefficient α of the 3D porous VN microparticles can be calculated using equation (1):

$$A = \alpha LC \tag{1}$$

In equation (1), *A* refers to the absorbance of 3D porous VN microparticles at 532 nm, *α* is the mass extinction coefficient of 3D porous VN microparticles (L g^{-1} cm^{-1}), *L* is the optical length of the quartz cuvette (cm), and *C* is the mass concentration (g L^{-1}). According to Figure S20a, the calculated mass extinction coefficient of 3D porous VN is 0.91 L g^{-1} cm^{-1}.

The photothermal conversion efficiency (*η*) of the 3D porous VN microparticles was determined by equation (2) as the previous reports[7]. The temperature change of the 3D porous VN aqueous solution (1 mg mL^{-1}) was recorded as a function of time under continuous irradiation of 532 nm laser at a power of 1 W cm^{-2}, in which the irradiation lasted for 600 s, and then the laser was shut off (Figure S20b).

$$\eta = \frac{\mathrm{h}S(T_{\max} - T_{sur}) - Q_{Dis}}{I(1 - 10^{-A532})} \tag{2}$$

Following the previous reports, the value of h*S* can be calculated by equation (3)-(5). The *T*, T_{max} and T_{sur} are random temperature, the maximum temperature after irradiation and the surrounding temperature. In this work, we measured the temperature change at concentration of 1 mg/mL, the $T_{max} - T_{sur}$ = 64.6 °C, and the τ_s was calculated to be 378.64 s by equation (3) and (4) as Figure S20b and S20c. m and C_p in equation (5) are the mass and heat capacity of solvent (water). In this work m = 1 g, C = 4.2 J g^{-1} °C. The hS value was determined to be 9.95 mW / °C.

$$\theta = \frac{T - T_{sur}}{T_{\max} - T_{sur}} \quad (3)$$

$$\mathrm{t} = \tau_s \ln \theta \quad (4)$$

$$\mathrm{h}S = \frac{\sum_i miC_p}{\tau_s} \quad (5)$$

Take the hS value into equation (2). At concentration of 1 mg/mL 3D porous VN, A_{532}=0.91. Q_{Dis} expresses the heat dissipated due to light absorption by the pure water without 3D porous VN. It is measured to be 12.89 mW. *I* is the incident laser power (1000 mW). Substituting these values into equation (2), the 633 nm laser photothermal conversion efficiency (η) of 3D porous VN can be calculated to be 67.3 %.

(2) Supplementary Figures

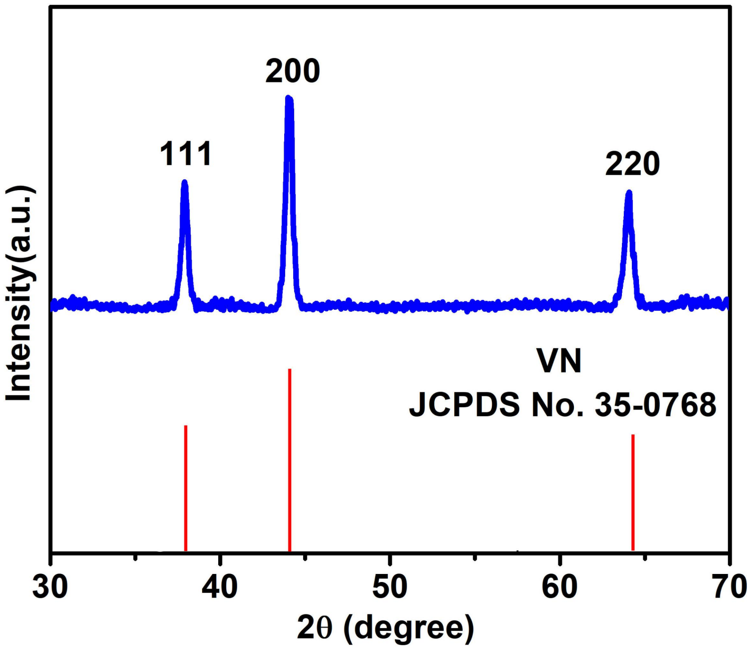

Supplementary Figure 1. XRD pattern of the as-synthesized 3D porous cubic phase VN sample.

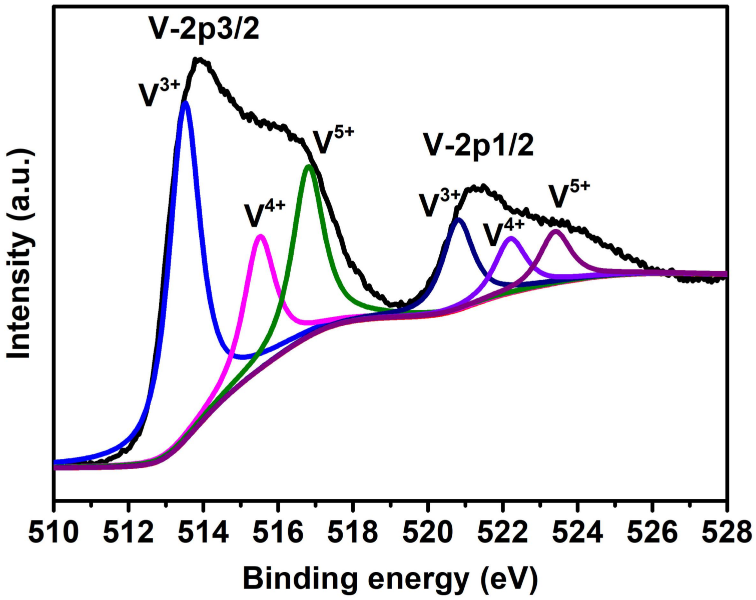

Supplementary Figure 2. XPS spectrum of the as-synthesized 3D porous VN sample.

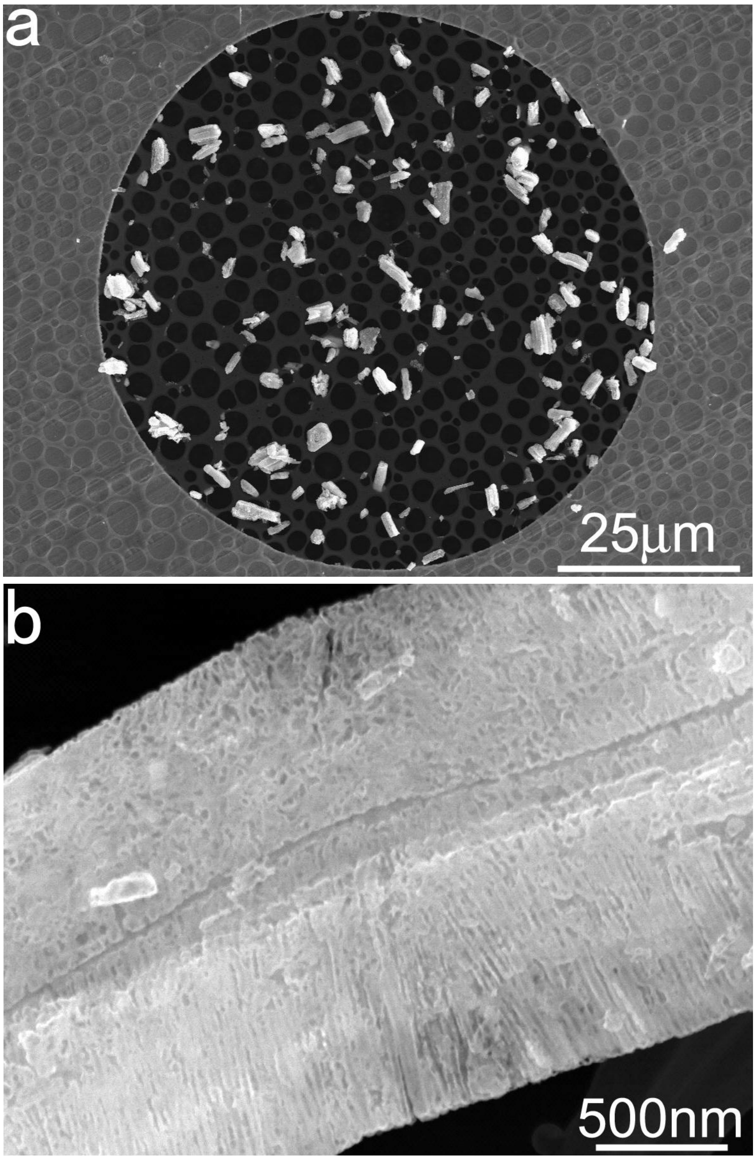

Supplementary Figure 3. (a) Large-scale browse image of the sample by low magnification SEM. (b) High-magnification SEM image of the sample.

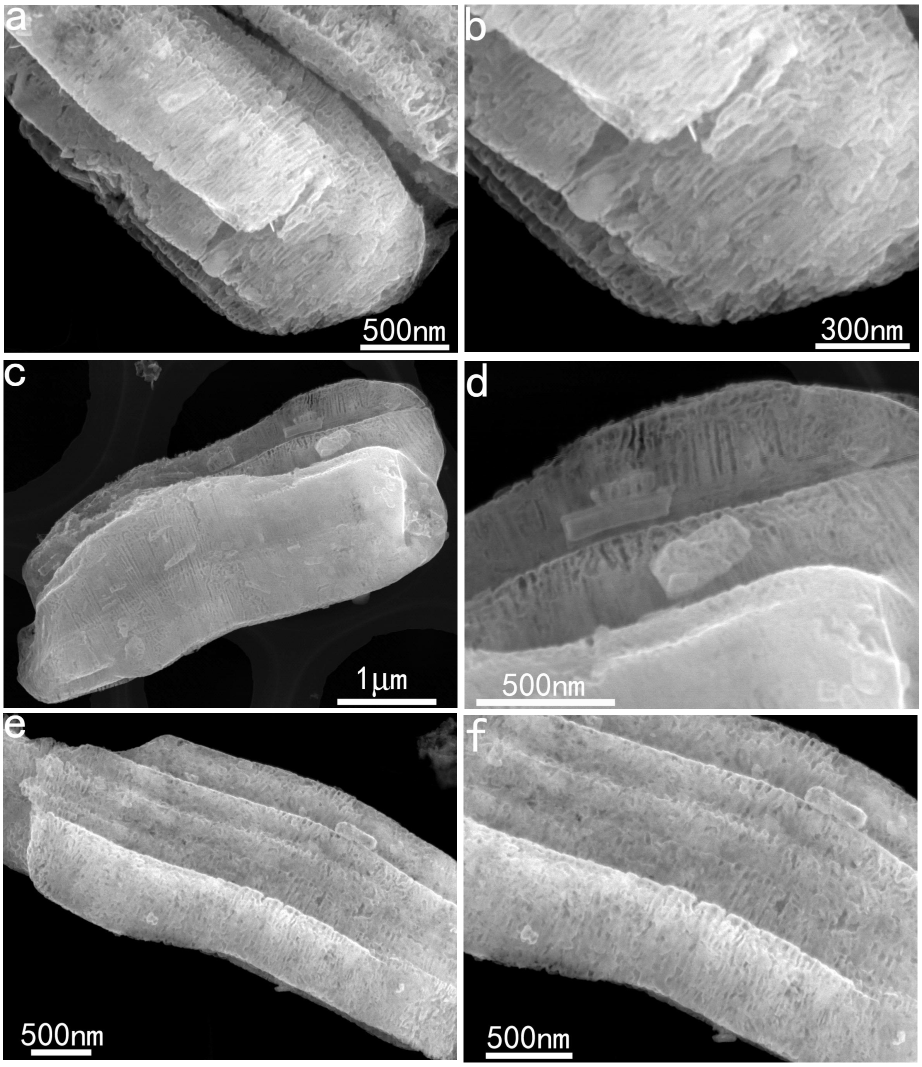

Supplementary Figure 4. More SEM images of the as-synthesized columnar 3D porous VN samples.

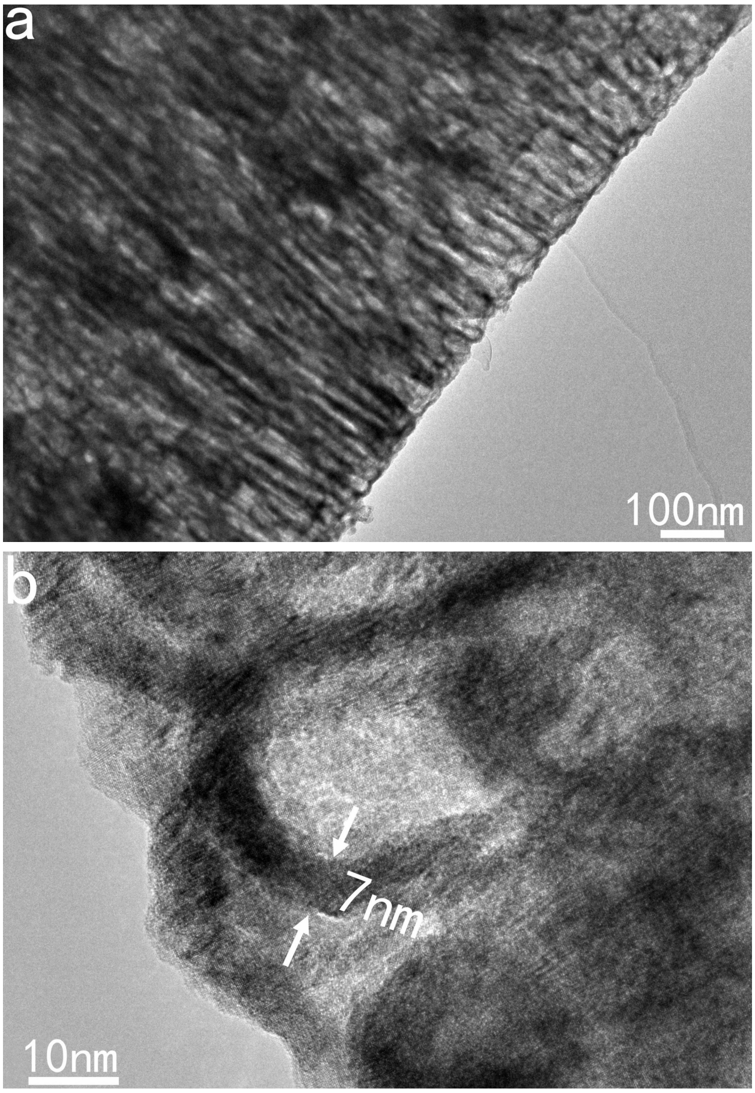

Supplementary Figure 5. High-magnification TEM images of the 3D porous VN sample, which shows that the thickness of the nanotube wall is about 6-8 nm.

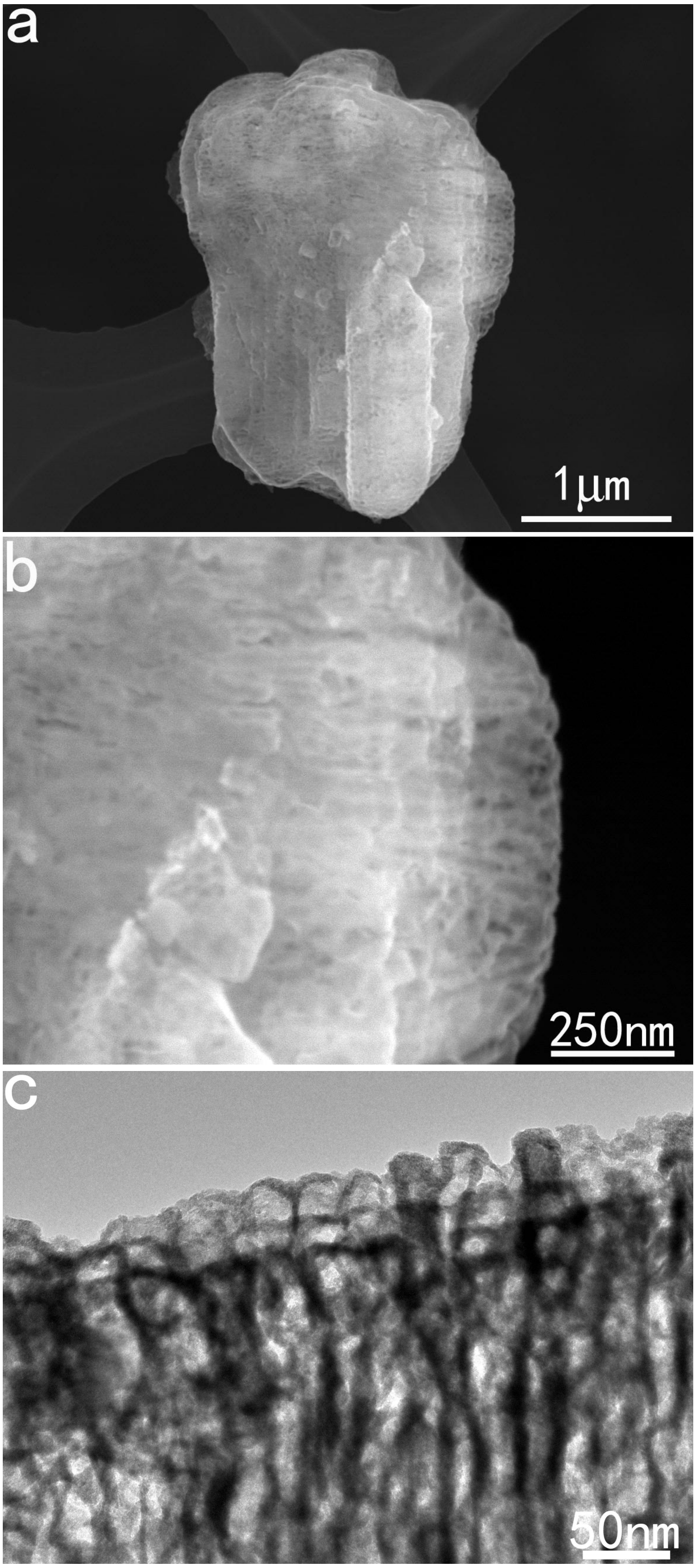

Supplementary Figure 6. Typical SEM and TEM images of the ellipsoid-like 3D VN samples.

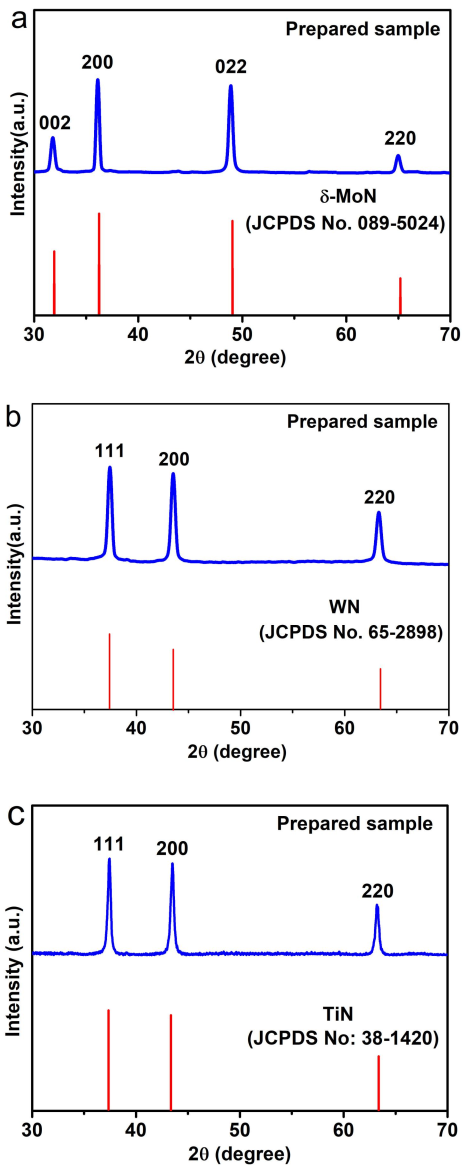

Supplementary Figure 7. XRD patterns of the as-synthesized 3D porous MoN (a), WN (b), and TiN (c).

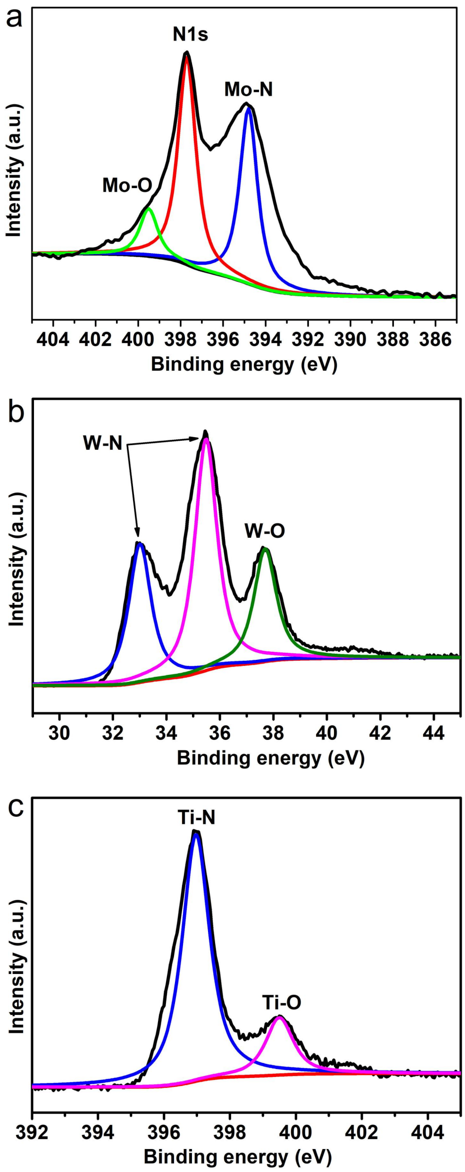

Supplementary Figure 8. XPS spectra of the as-synthesized 3D porous MoN, WN, and TiN.

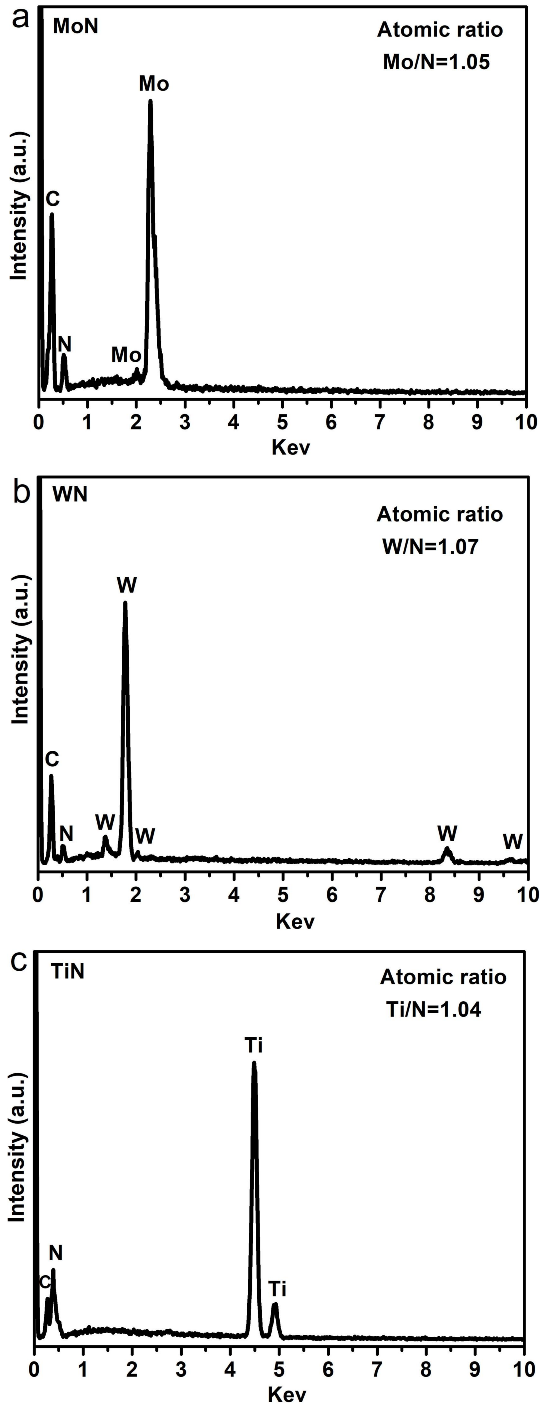

Supplementary Figure 9. EDS spectrum of the as-synthesized 3D porous MoN, WN, and TiN.

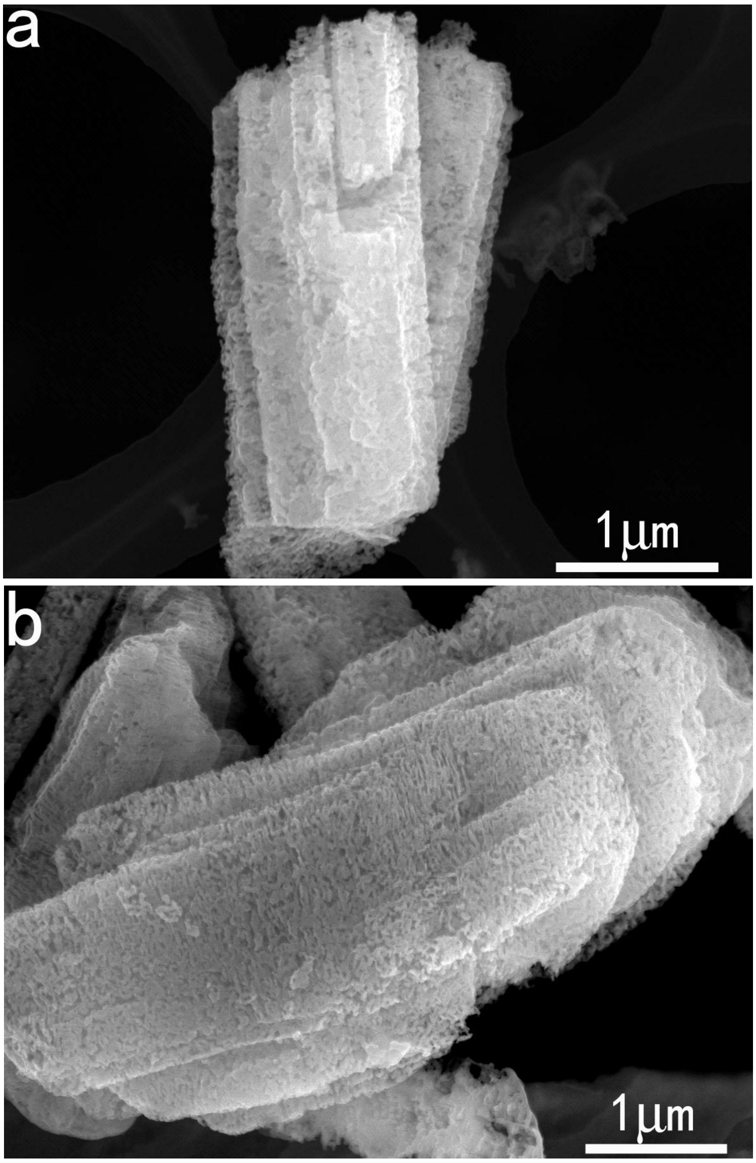

Supplementary Figure 10. SEM images of 3D porous ZnO microparticles obtained at 290 °C after 0.5 h.

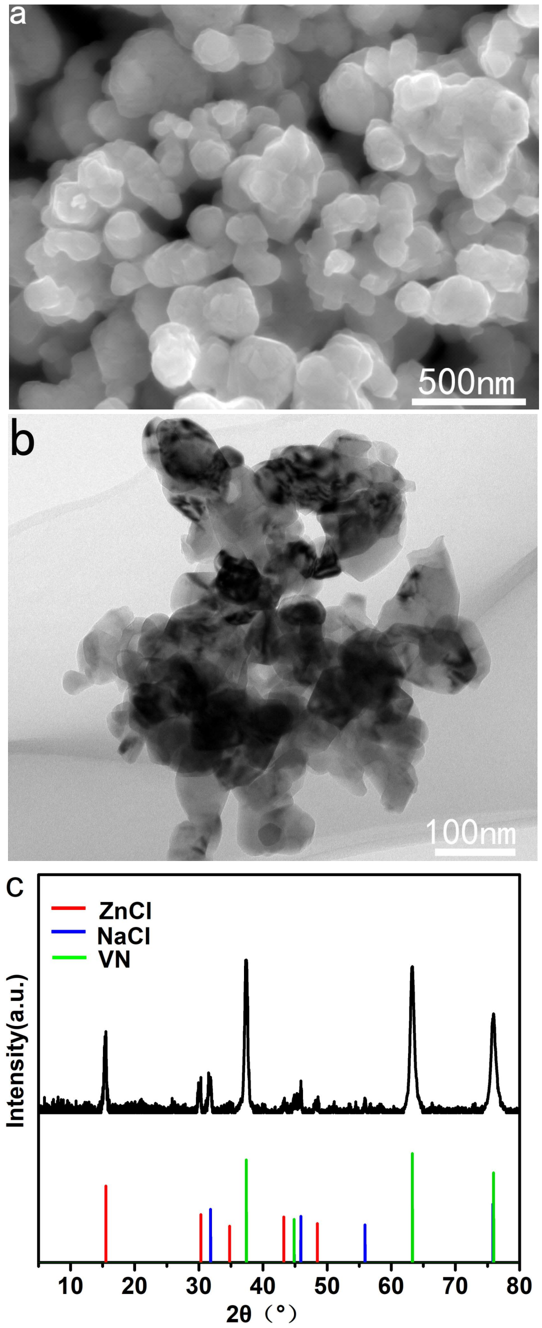

Supplementary Figure 11. SEM (a) and TEM (b) images of the VN particles prepared when $ZnCl_2 \cdot 6H_2O$ is absent, which shows that the resulting product is irregular solid particles. (c) XRD pattern of the sample before acid washing, which indicates that it is a mixture of VN, $ZnCl_2$, and NaCl.

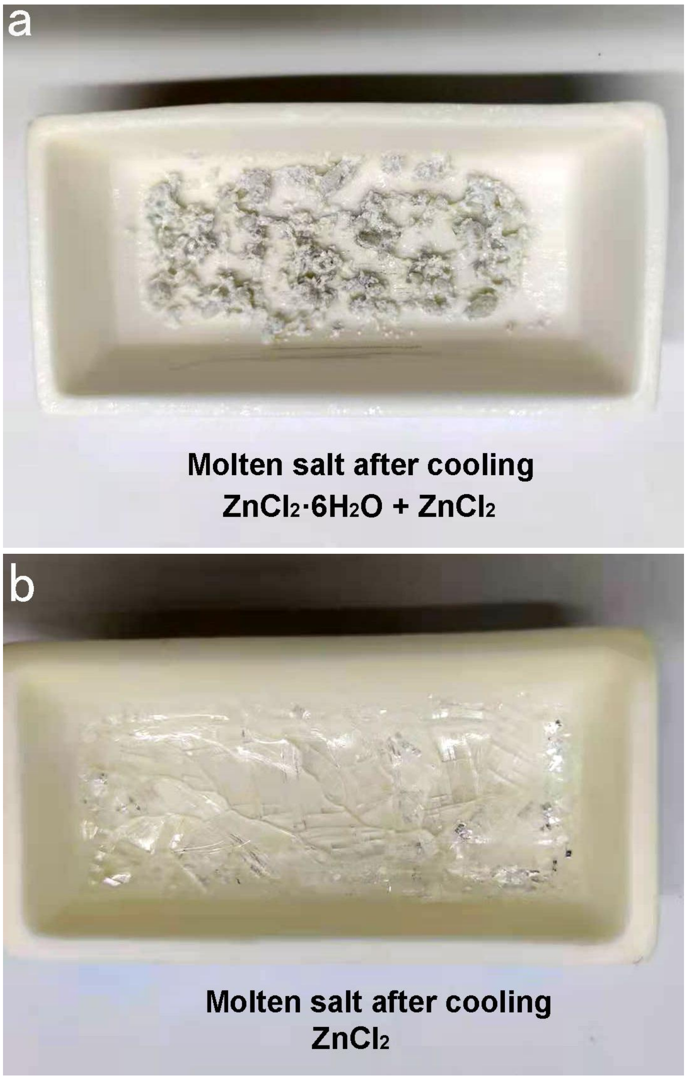

Supplementary Figure 12. SEM images of the molten salt after cooling. (a) The precursor of $ZnCl_2 \cdot 6H_2O$ and $ZnCl_2$ (mass ratio is 1:20) was transformed into the mixture of ZnO and $ZnCl_2$ during the melting process, and a large number of holes are formed. (b) When the molten salt is only $ZnCl_2$, the molten salt is very transparent and smooth after cooling, and no holes are formed.

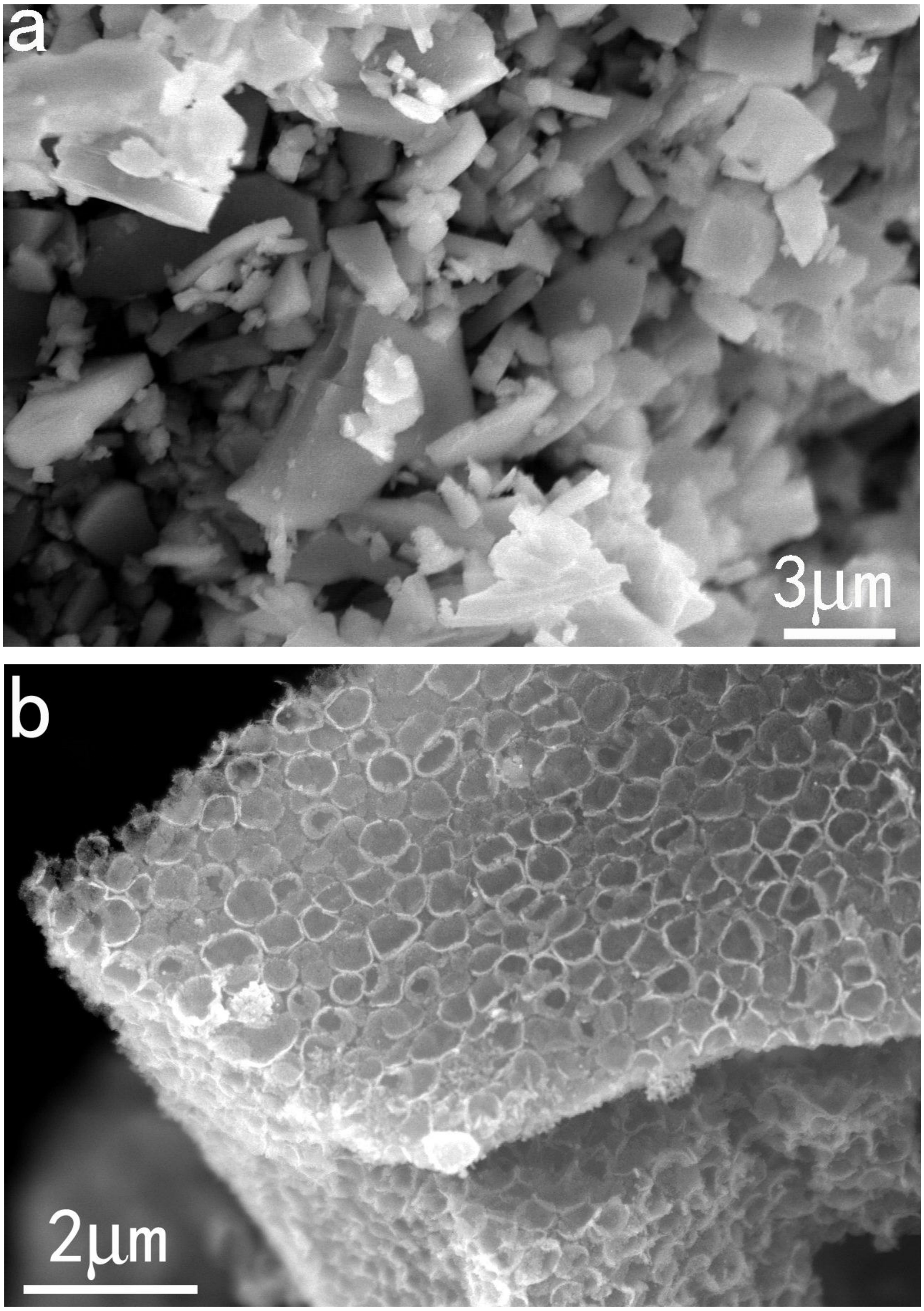

Supplementary Figure 13. SEM images of VN samples obtained under different $ZnCl_2 \cdot 6H_2O$ contents. (a) 0.5 g. (b) 6g. In these comparative experiments, the quality of $ZnCl_2$ is 40 g.

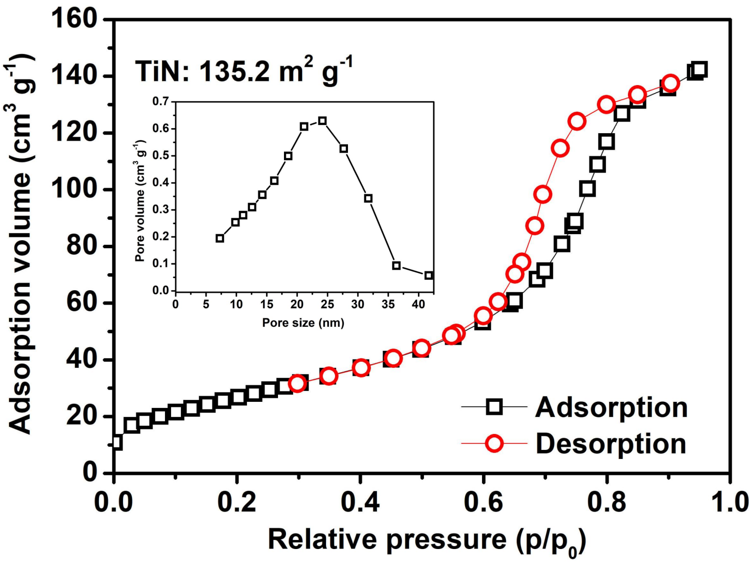

Supplementary Figure 14. N_2 adsorption and desorption isotherms of the 3D porous TiN, inset: pore size distribution.

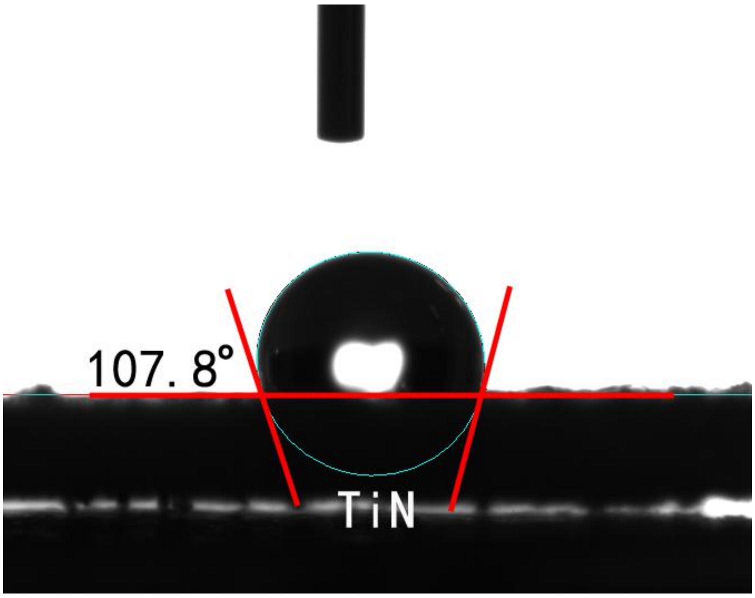

Supplementary Figure 15. Contact angle of the 3D porous TiN film measured at the interface of water drop and film.

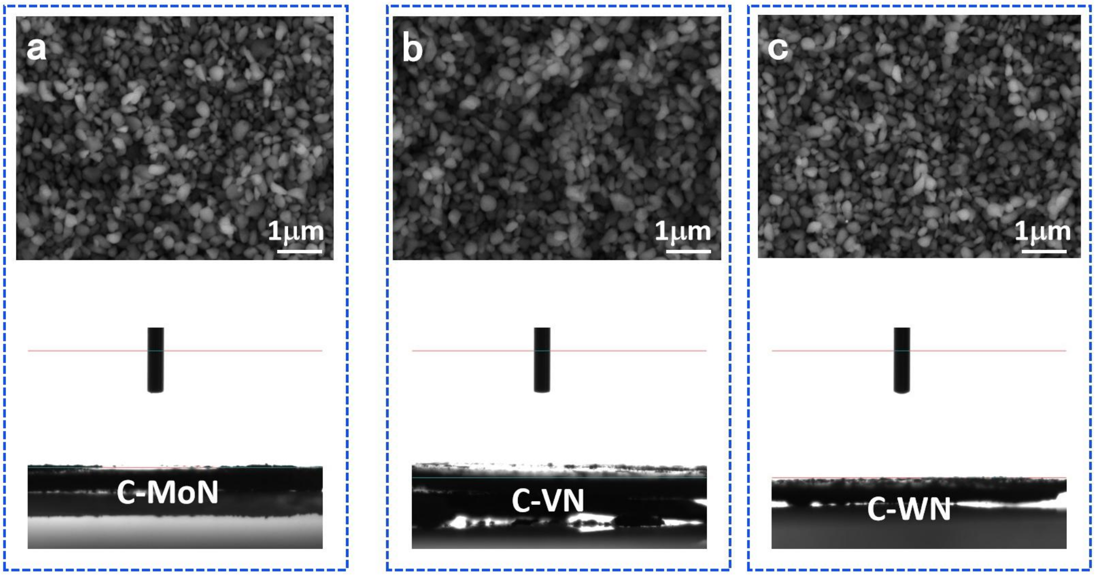

Supplementary Figure 16. The SEM images of the commercial C-VN, C-MoN, and WN samples and their corresponding water contact angles.

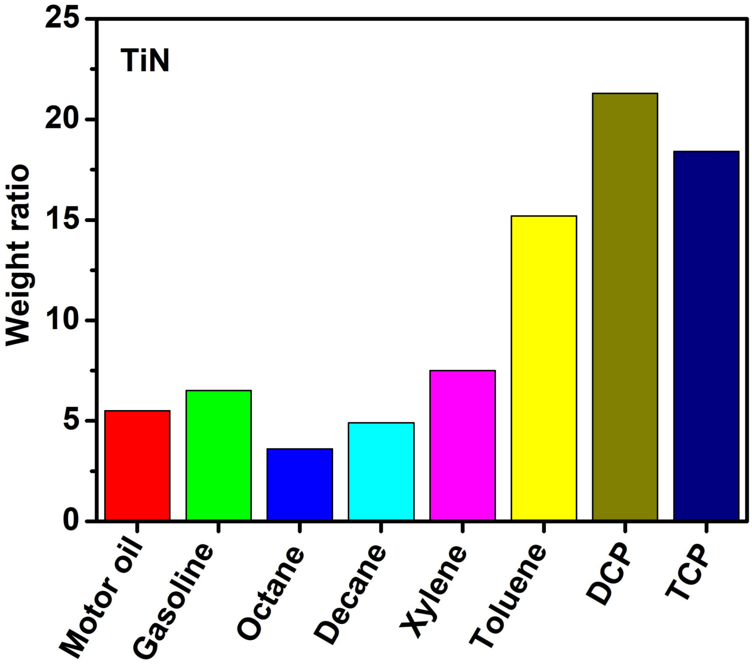

Supplementary Figure 17. Saturated adsorption capacity of TiN for organic compounds.

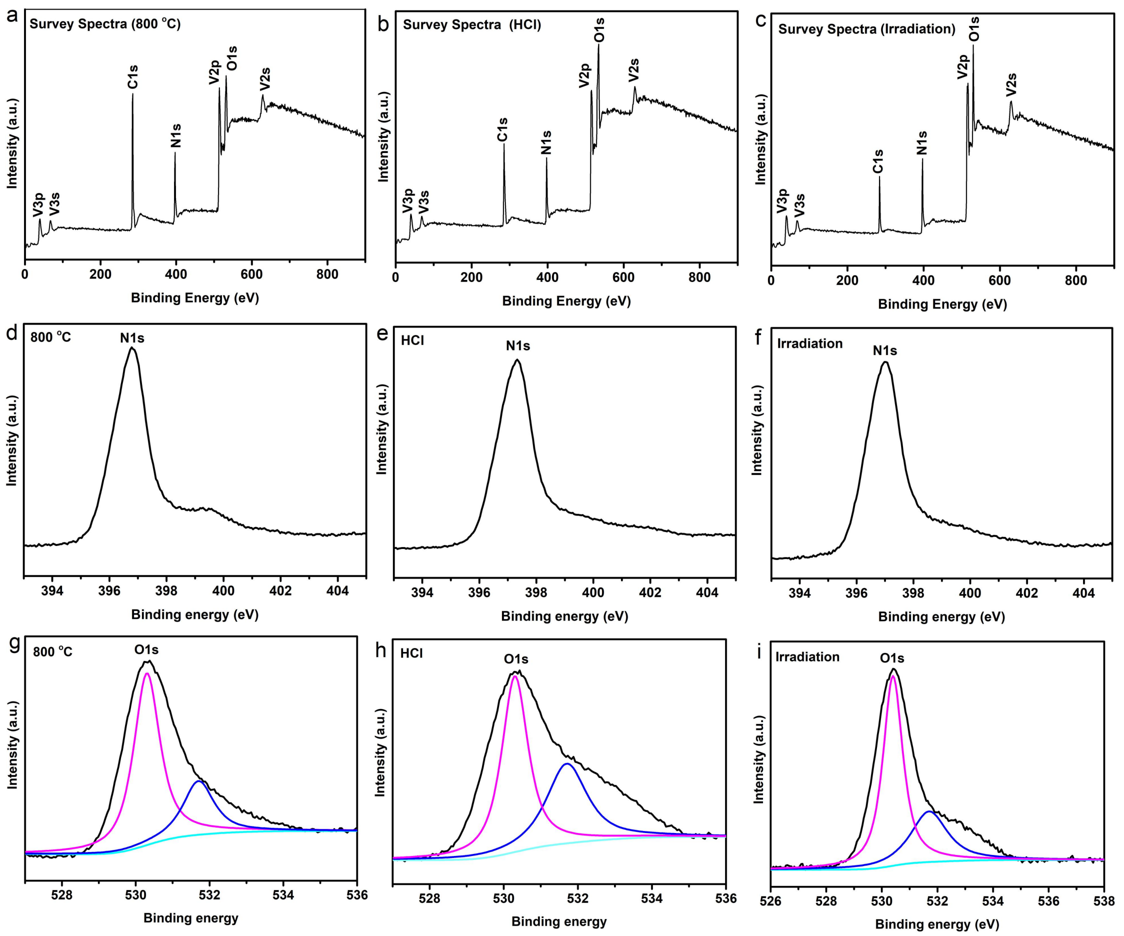

Supplementary Figure 18. XPS characterization of the VN samples after various treatment. (a-c) Survey spectra. (d-f) N1s spectra. (g-i) O1s spectra. It can be seen from these spectra that the surface states of these VN samples remain almost unchanged after various treatments. It should be noted that the clear O1s line confirms that a thin oxide layer exists on the surface of VN. The oxygen signal is fitted with two peaks. The main component is centered at 530.3 eV and is typical for oxygen in a metal oxide. The second small peak is at about 531.7 eV and can be attributed to the signal from -OH groups chemisorbed at the surface.

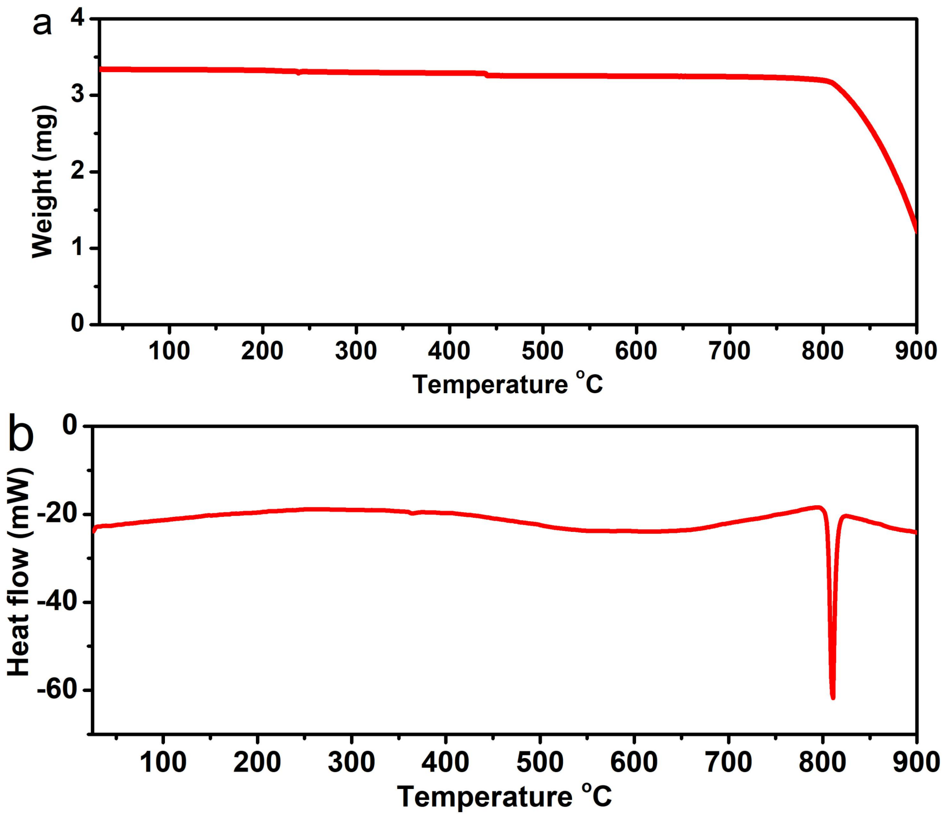

Supplementary Figure 19. Thermal stability analysis of 3D porous VN in N_2. (a) TGA curve. (b) DSC curve. From the results of TG and DSC, VN sample is stable below 800 °C in N_2 atmosphere.

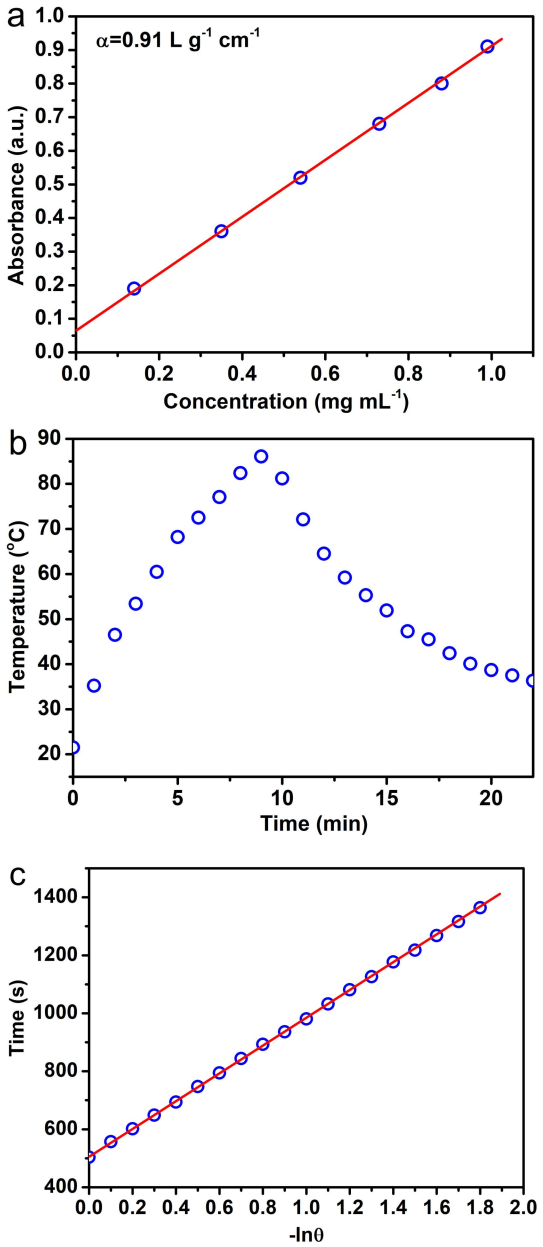

Supplementary Figure 20. (a) The linearly fitted plots of absorbance versus concentration of 3D porous VN aqueous suspension at 532 nm. (b) Photothermal effect of aqueous dispersion of the 3D porous VN (1 mg/mL) under irradiation with the laser (532 nm, 1 W/cm^2) for one on/off cycle. (c) Time constant for heat transfer from the system is determined to be τs= 363.25 s by applying the linear time data from the cooling period (after 600 s) versus negative natural logarithm of driving force temperature, which is obtained from the cooling stage of (b).

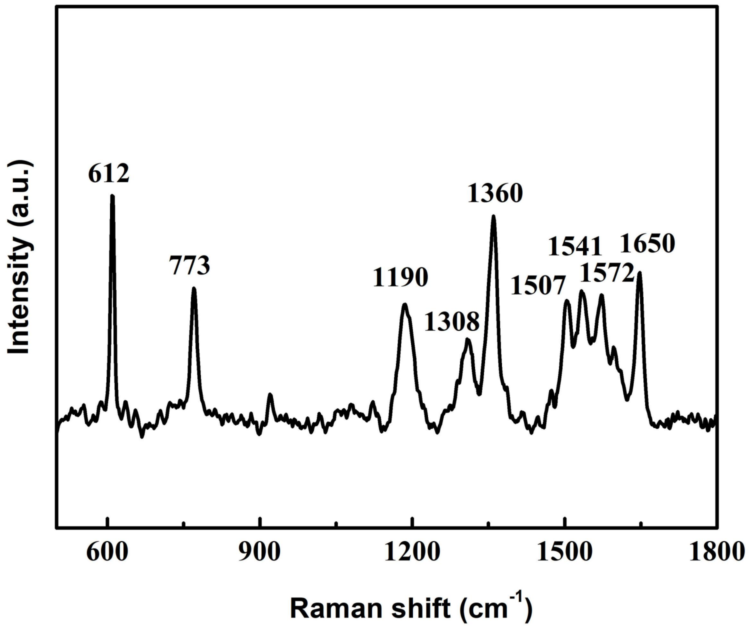

Supplementary Figure 21. The standard Raman spectrum of R6G reference material.

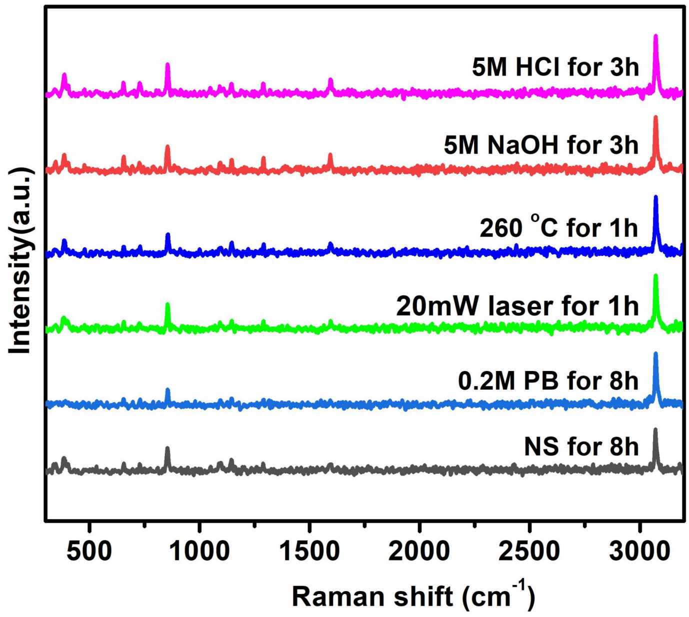

Supplementary Figure 22. The Raman spectra of 2,5-DCP obtained on 3D porous VN after various treatments. Laser power: 0.5 mW; Integration time: 60 s.

(3) Supplementary Table 1: some of the previously reported EFs for typical non-noble Metal Raman substrate materials

Substrate	Probe molecule	Excited wavelength (nm)	Author	EF	Stability
TiO_2 photonic microarray	MB	532	D. Qi et al.[8]	2×10^4	stable
CdTe nanoparticles	4-Mpy	514.5	Y. F. Wang et al.[9]	10^4	Liable to oxidation and corrosion
ZnO nanoparticles	D266	488	H. Wen et al.[10]	50	Liable to corrosion
CdS nanoparticles	4-Mpy	514.5	Y. F. Wang et al.[11]	10^2	Liable to oxidation and corrosion
$\alpha - Fe_2O_3$	4-Mpy	514.5	X. Q. Fu et al.[12]	2.7×10^4	Liable to corrosion
Cu_2O	4-MBA	488	L. Jiang et al.[13]	10^5	Liable to oxidation and corrosion
CuO nanoplates	4-Mpy	514.5	Y. Wang et al.[14]	10^2	Liable to corrosion
$W_{18}O_{49}$ nanorods	R6G	532.8	S. Cong et al.[15]	3.4×10^5	Liable to oxidation
Cu_2O superstructure	R6G	532	L. Guo et al.[16]	8×10^5	Liable to oxidation and corrosion
MoO_2 nanodumbbell	R6G	532	G. C. Xi et al.[17]	1.2×10^6	stable
MoS_2 nanosheet	R6G	532.8	Zheng, Z. H. et al.[18]	1.6×10^5	Liable to oxidation
MOF	R6G	R6G 532.8	Sun, H. Z. et al.[19]	10^6	Liable to oxidation

					and corrosion
Organic Semiconductor	DFH-4T	532	Yilmaz M. et al.[20]	3.4×10^3	Liable to oxidation and corrosion
Nb_2O_5 nanorods	MB	532	Shan, Y. F. et al.[21]	7.1×10^6	stable
Amorphous ZnO Nanocages	4-MBA	633	Wang, X. T. et al.[22]	6.6×10^5	Liable to corrosion
Amorphous TiO_2 nanosheets	4-MBA	633	Wang, X. T. et al.[23]	1.8×10^6	stable
**VN (the present work)**	**R6G**	**532**	**Guan, H. M. et al.**	**6.2×10^7**	**stable**

(4) Supplementary References

1. G. Kresse, J. Furthmüller, *Comput. Mater. Sci.* **1996**, *6*, 15-50.

2. G. Kresse, J. Furthmüller, *Phys. Rev. B* **1996**, *54*, 11169-11186.

3. J. P. Perdew, K. Burke, M. Ernzerhof, *Phys. Rev. Lett.* **1996**, *77*, 3865-3868.

4. G. Kresse, D. Joubert, *Phys. Rev. B* **1999**, *59*, 1758-1775.

5. H. J. Monkhorst, J. D. Pack, *Phys. Rev. B* **1976**, *13*, 5188-5192.

6. C. Stampfl, W. Mannstadt, R. Asahi, A. J. Freeman, *Phys. Rev. B* **2001**, *63*, 155106.

7. L. Yuwen, J. Zhou, Y. Zhang, Q. Zhang, J. Shan, Z. Luo, L. Weng, Z. Teng, L. Wang, *Nanoscale* **2016**, *8*, 2720-2726.

8. Qi, D., Lu, L., Wang, L.& Zhang, J. Improved SERS sensitivity on plasmon-free TiO_2 photonic microarray by enhancing light-matter coupling. *J. Am. Chem. Soc.* **136,** 9886-9889 (2014).

9. Wang, Y. F. et al. Surface-enhanced Raman scattering on nercaptopyridine capped CdS microclusters. *Spectrochim*. *Acta*, *Part A* **66**, 1199–1203 (2007).

10. Wen, H., He, T. J., Xu, C. Y., Zuo, J. & Liu, F. C. Surface enhancement of Raman and absorption apectra from cyanine dye D266 adsorbed on ZnO colloids. *Molecular Physics*. **88**, 281–290 (1996).

11. Wang, Y. F. et al. Mercaptopyridine surface-functionalized CdTe quantum dots with enhanced Raman scattering properties. *J. Phys. Chem. C*. **112**, 996–1000 (2008).

12. Fu, X. Q., Bei, F. L., Wang, X., Yang, X. J. & Lu, L. D. Surface-enhanced Raman scattering of 4-mercaptopyridine on sub-monolayers of α-Fe_2O_3 nanocrystals (sphere, spindle, cube). *J. Raman Spectrosc.* **40**, 1290–1295 (2009).

13. Jiang, L. et al. Surface-enhanced Raman scattering spectra of adsorbates on Cu_2O nanospheres: charge-transfer and electromagnetic enhancement. *Nanoscale* **5**, 2784–2789 (2013).

14. Wang, Y. et al. Enhanced Raman scattering as a probe for 4-mercaptopyridinesurface-modified copper oxide nanocrystals. *Anal. Sci.* **23**, 787–791 (2007).

15. Cong, S. et al. Noble metal-comparable SERS enhancement from semiconducting metal oxides by making oxygen vacancies. *Nat. Commun*. **6**, 7800 (2015).

16. Lin, J., Shang, Y.; Li, X. X.; Yu, J. Wang, X. T. & Guo, L. Ultrasensitive SERS detection by defect engineering on single Cu_2O superstructure particle. *Adv. Mater.* **29**, 1604797 (2017).

17. Zhang, Q. Q. et al. A metallic molybdenum dioxide with high stability for surface enhanced Raman spectroscopy. *Nat. Commun.* **8**, 14903 (2017).

18. Zheng, Z. H. et al. Semiconductor SERS enhancement enabled by oxygen incorporation. *Nat. Commun.* **8**, 1993 (2017).

19. Sun, H. Z. et al. Metal-organic frameworks as surface enhanced Raman scattering substrates with high tailor ability. *J. Am. Chem. Soc.* **141**, 870-878 (2019).

20. Yilmaz M. et al. Nanostructured organic semiconductor films for molecular detection with surface-enhanced Raman spectroscopy. *Nat. Mater.* **16**, 918-924 (2017).

21. Shan, Y. F. et al. Niobium pentoxide: a promising surface-enhanced Raman scattering active semiconductor substrate. *npj Comput. Mater.* **3**, 11 (2017).

22. Wang, X. T. et al. Remarkable SERS activity observed from amorphous ZnO nanocages. *Angew. Chem. Int. Ed.* **56**, 9851-9855 (2017).

23. Wang, X. T. et al. Two-dimensional amorphous TiO_2 nanosheets enabling high-efficiency photoinduced charge transfer for excellent SERS activity. *J. Am. Chem. Soc.* **141**, 5856-5862 (2019).
